# Developing Effective Methods for Electronic Health Personalization: Protocol for Health Telescope, a Prospective Interventional Study

**DOI:** 10.2196/16471

**Published:** 2020-07-31

**Authors:** Bastiaan Johannes Paulus Cornelis Willemse, Maurits Clemens Kaptein, Fleur Hasaart

**Affiliations:** 1 Jheronimus Academy of Data Science 's-Hertogenbosch Netherlands; 2 Department of Medical and Clinical Psychology Tilburg University Tilburg Netherlands; 3 CZ Zorgverzekeraars Tilburg Netherlands

**Keywords:** eHealth, mHealth, personalization, longitudinal study, wearables, panel study, persuasive technology, gdpr

## Abstract

**Background:**

Existing evaluations of the effects of mobile apps to encourage physical activity have been criticized owing to their common lack of external validity, their short duration, and their inability to explain the drivers of the observed effects. This protocol describes the setup of Health Telescope, a longitudinal panel study in which the long-term effects of mobile electronic health (eHealth) apps are investigated. By setting up Health Telescope, we aim to (1) understand more about the long-term use of eHealth apps in an externally valid setting, (2) understand the relationships between short-term and long-term outcomes of the usage of eHealth apps, and (3) test different ways in which eHealth app allocation can be personalized.

**Objective:**

The objectives of this paper are to (1) demonstrate and motivate the validity of the many choices that we made in setting up an intensive longitudinal study, (2) provide a resource for researchers interested in using data generated by our study, and (3) act as a guideline for researchers interested in setting up their own longitudinal data collection using wearable devices. For the third objective, we explicitly discuss the General Data Protection Regulation and ethical requirements that need to be addressed.

**Methods:**

In this 4-month study, a group of approximately 450 participants will have their daily step count measured and will be asked daily about their mood using experience sampling. Once per month, participants will receive an intervention containing a recommendation to download an app that focuses on increasing physical activity. The mechanism for assigning recommendations to participants will be personalized over time, using contextual data obtained from previous interventions.

**Results:**

The data collection software has been developed, and all the legal and ethical checks are in place. Recruitment will start in Q4 of 2020. The initial results will be published in 2021.

**Conclusions:**

The aim of Health Telescope is to investigate how different individuals respond to different ways of being encouraged to increase their physical activity. In this paper, we detail the setup, methods, and analysis plan that will enable us to reach this aim.

**International Registered Report Identifier (IRRID):**

PRR1-10.2196/16471

## Introduction

### Background

The World Health Organization has identified physical inactivity as the fourth leading risk factor of death worldwide [1]. Physical inactivity is defined as *the absence of sufficient bodily movement produced by skeletal muscles requiring energy expenditure*. It has been estimated that every year, over 5 million people die as a result of insufficient physical activity. Physical inactivity carries part of the burden of, among others, coronary heart disease, type 2 diabetes, breast cancer, and colon cancer, as one of the multiple causes of these diseases. Additionally, increased physical inactivity affects mental health, with research showing a positive correlation between physical inactivity and depression [2]. Research estimates that an increase of 10% in activity can save 1.3 million lives yearly [3].

In this paper, we describe the protocol of Health Telescope, a unique longitudinal study researching the effects of the personalized offering of various existing electronic health (eHealth) apps designed to motivate users to increase physical activity. In recent years, longitudinal studies have been argued to be unavoidable for research investigating behavior change, as solidifying behavior change may take months or years [4]. We took up this challenge of measuring the effects of behavior change apps over a long period of time. Furthermore, we specifically focus on the personalized offering of these apps to investigate whether personalization can improve the effectiveness of common eHealth apps. This protocol paper describes the setup, methods, materials, and analysis plan of Health Telescope to enable other researchers to understand and judge the validity of the data collected within the project. In our description, we focus strongly on the ethical and legal aspects of setting up our panel study. We hope this paper provides a useful resource for others aiming to set up longitudinal eHealth studies.

### The Rise and Effects of eHealth

Mobile apps aimed at making users improve their health autonomously have been growing in number in recent years, including apps that persuade users to be more active, apps that track food intake, and apps that offer help with mental health issues. The total value of this mobile health or eHealth app market was estimated in 2018 at US $28.32 billion and is projected to reach US $102.35 billion by 2023 [5]. These apps have the potential to increase the level of control that individuals have over their health while improving general health in several ways. Among other factors, users may identify health issues earlier, autonomous use can be a lower barrier than receiving help from a professional, and new routes for preventative measures can be taken.

The number of mobile apps labeled as eHealth apps is growing tremendously, with over 1000 new apps entering top app stores every day [6]. This increase is accompanied by a growing skepticism, as research has not yet conclusively shown a positive effect on health and long-term wellbeing [7-9]. The absence of a convincing effect of eHealth apps is largely attributed to nonusage [10], which might itself be caused by distinct characteristics of eHealth services [10], social aspects of use [10], and eHealth literacy [11].

The rapid proliferation and short lifetime of apps make identifying apps that accomplish the intended behavior change effectively for a group of users a very difficult task. Properly identifying behavior change is not trivial, as can be demonstrated by a simple example as follows. Suppose an individual adopts the mindset of changing ways and being healthier, and in this process, downloads an eHealth app. After using the app for several weeks, the app is abandoned. However, over the course of months, the user does change ways. This change can, at least in part, be attributed to the earlier usage of the eHealth app. This scenario demonstrates that it is firstly difficult to determine whether an app contributes to a change in lifestyle and that it is secondly difficult to measure the outcome if the user has stopped engaging with the app.

Additionally, as has been demonstrated before in the literature [12], the effectiveness of eHealth apps to motivate healthy behaviors might, in part, be driven by a correct match between the app itself and its user (ie, a personalized app might have a larger effect). One can wonder whether currently, the apps that users download from the various app stores provide a correct match. All of the major app stores offering eHealth apps currently use a 5-star rating system to grade their apps, which is one of the factors driving the download behaviors of users. However, these ratings arguably have their issues [13,14]. While alternatives have been proposed [15], we currently do not properly understand how we can personalize the choice of eHealth apps [16]. Recently, there has been substantial interest in sequential allocation methods that combine machine learning to predict the effects of treatments for individuals based on historical data, with effective methods to balance exploring different treatments and exploiting the seemingly best treatment [17]. Effectively, these novel methods select personalized treatments as data are collected over time. This is a potentially promising approach as it allows us to reach beyond simple user ratings by using data to improve the match between a user and an app.

### Health Telescope

To answer these pressing questions regarding the effects (both long-term and short-term effects) and methods for personalization of eHealth apps, we set up Health Telescope, a large-scale interventional panel study. In the current protocol paper, we highlight the choices we made in setting up this prospective study. Health Telescope is used to actively and iteratively test different approaches of personalization and, at the same time, track the longitudinal effectiveness of these personalized offerings. Health Telescope allows us to answer major open questions regarding the effectiveness of eHealth apps. First, by closely monitoring app usage, engagement, activity, and mood, we aim to obtain a better understanding of the short-term and long-term effects of eHealth apps. Second, by actively experimenting with different eHealth app encouragement schemes, we test the effects of personalizing eHealth offerings.

The importance of large-scale studies that follow individuals for a longer period has been argued in recent years [18]. To our best knowledge, Health Telescope is the first large-scale long-term study that follows and intensively gathers activity and behavioral data from participants. With this protocol, we aim to motivate researchers to set up similar studies, as we believe research in eHealth stands to benefit from thorough long-term studies.

The Health Telescope study aims to achieve the following three objectives, labeled study objectives 1-3 (SOs 1-3):

- SO1: The study aims to measure the effect of eHealth for longer periods. To accomplish this, we choose a panel uptime of at least 4 months.
- SO2: The study aims to correlate short-term and long-term measures. To accomplish this, we combine behavioral measures with periodic surveys and analyze their relations over time.
- SO3: The study aims to test the effects of personalization in allocating health apps. To accomplish this, we set up iterative interventions and an allocation scheme that uses collected data to predict which health app is expected to lead to the highest activity increase, given a person’s background information, activity, and behavior.

In this protocol description paper, we aim to accomplish the following three objectives, labeled protocol objectives 1-3 (POs 1-3):

- PO1: The paper aims to demonstrate and motivate the validity behind the setup of Health Telescope.
- PO2: The paper aims to confirm its function as a steppingstone for future researchers interested in the data generated by Health Telescope.
- PO3: The paper aims to serve as a guideline for those setting up longitudinal studies using wearable devices.

The rest of the paper is structured as follows: the Methods section and its various subsections elaborate on the research questions and describe the various aspects in setting up the panel; the Results section briefly expands on the timeline of the project; and the Discussion section goes into the advantages and disadvantages of the chosen setup and presents the conclusions.

## Methods

### Study Design and Aims

Health Telescope is a prospective interventional panel study (N=450) measuring activity and iteratively testing the effect of recommending distinct eHealth apps to participants, with the goal of personalization (ie, finding a relation between an individual and an app that can provide motivation to be sufficiently active). The study is designed to run a minimum of 4 months. We would like to continue to monitor participants past these 4 months to further measure the long-term effects of eHealth app usage. We plan to do so assuming the dropout rate of participants is low enough to keep a sufficiently large group for further analysis.

The study has been approved by METCBrabant, the Ethics Review Board of Tilburg University, the Netherlands, and the General Data Protection Regulation (GDPR) compliance officer of Tilburg University. Furthermore, a Data Protection Impact Assessment check was performed by the Technical University of Eindhoven and Tilburg University. We detail the approval process in the sections “Ethical Approval” and “GDPR Compliance” to aid researchers who, like us, have to go through this process (PO3).

During the study, participant data are measured, and participants receive a daily request to complete a three-question survey concerning their mood (this is detailed in the section “Health Telescope App”). The set of interventions is defined as five messages that will be sent to participants’ smartphones, four of which contain a recommendation to download a specific health app (we expand upon this in the section “Interventional Apps”), and the fifth does not recommend any app. The messages recommending apps provide a brief textual summary of the app’s functionalities. Every month, each participant will be allocated to one of the interventions.

The allocation of interventions is, in part, personalized. We detail our personalization logic in the section “Allocation of Interventions” below.

The participants will be recruited in Q4 of 2020 (details are provided in the section “Recruitment”) and are expected to participate for a minimum of 4 months. The participant group of the panel will be made up of a diverse set of Dutch adults who are interested in using mobile health apps, recruited mainly through general practitioners (more information is provided in the “Recruitment” section). We select this wide range of users to allow the results to speak for a general audience who may download a mobile health app (which helps answer SO1 and SO3). It is relevant to mention that, to appeal to the participant group, the apps chosen to be recommended in the panel (detailed in the section “Interventional Apps”) do not require a user to be in superior physical shape for use.

Data are collected through a combination of the following: a wearable device handed out to participants at the start of the study, a mobile app that tracks phone use and GPS, and survey responses measuring mood and happiness using the experience sampling method (ESM) [19].

The section “Data Collection” provides a concrete overview of the data that are collected in the study. This diverse set of data collection methods allows us to monitor eHealth app usage and its effects on various outcome measures (SO1). Furthermore, the duration of the panel allows us to examine the relationship between the long-term and short-term measures of the effects of eHealth (SO2). Finally, we will provide interventions, with an intervention constituting a recommendation to start using a selected eHealth app. These interventions will, in part, be personalized (ie, we will recommend an app that we expect to be the most effective for a given user based on the data collected thus far). This personalization allows us to evaluate the effect of personalization (SO3).

### Recruitment

#### Inclusion Criteria

Participants are recruited via several paths. To be eligible for participation, a respondent needs to meet the following criteria: live in the Netherlands; speak Dutch; be 18 years or older; possess an Android phone running Android 6.0 or newer that they are willing to use in the study; be sufficiently smartphone and internet literate to use the app; and intend to participate for the full duration of the study.

Note that participants are considered smartphone and internet literate when they can navigate an Android smartphone and get through the setup process, including the introduction survey, app installation, and wearable setup. To ensure participants can perform these steps, the emphasis is put on highlighting the steps in the setup process during the information sessions that participants go through before entering the panel.

We deliberately do not focus on a specific health group, such as those defined by specific choices of BMI, chronic disease, or age. This is to ensure that knowledge gained from this project can be applied to the general group of app users (the group that downloads apps like the ones tested in this project). The panel will be formed through the following channels:

- Health insurance employees (approximately 150 participants): A large health insurer in the Netherlands that helped fund the Health Telescope project has indicated they would like to give their employees the chance to enroll in the panel. Advertisements will be sent out through internal communication channels, inviting employees to attend an introductory meeting where the purpose and details of the panel will be explained.
- General practitioner visitors (approximately 300 participants): Part of the panel will consist of individuals recruited directly through general practitioners. Three general practitioner offices in Geldrop, a village in Noord-Brabant with 28,500 inhabitants, have agreed to assist in recruitment. Advertising will be done in the practices, as well as through email.

#### Recruitment Process and Materials

**Figure 1:**
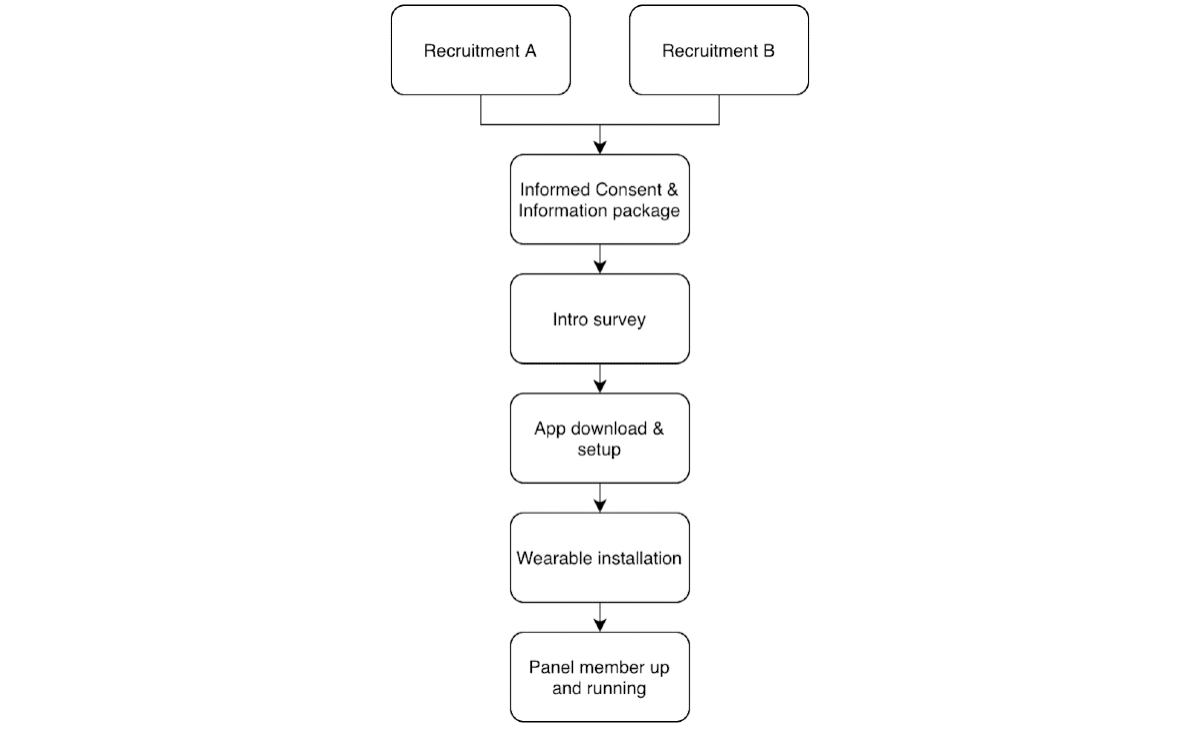
Steps in the enrollment of participants. Starting with recruitment (from different sources), participants receive study information and an informed consent (IC) form and take the introduction survey before downloading and installing the app. After installing the wearable, participants are set up and enrolled in the study.

To ensure participants understand the objectives of the study and the actions they are expected to perform, we set up information sessions that potential participants need to attend before entering the panel. In these sessions, the study goals and the steps of the setup that participants need to go through (detailed in Figure 1) are explained to a group of approximately 25 respondents.

Before entering the participant group, all respondents are given the necessary introductory documents. Included in this is an informed consent document that is mandatory for participation. These documents are available elsewhere [20]. If the respondents have additional questions before deciding to participate, they are able to pose them through the project website or by directly contacting the team through email. To ensure users understand what happens to their data, there is a thorough privacy policy available elsewhere [21].

Figure 1 shows the steps potential participants go through in the recruitment process. The individual steps are further described in the section “Data Collection.”

### Setup for Investigating Objectives

In setting up the study, we look at how to achieve the goals of the study as presented below.

#### SO1: Effects of eHealth Apps

To test the effects of eHealth apps, we will create a group of participants who receive a recommendation to use one of the interventional apps and compare this to a group of users who are not given this recommendation. Specifically, we will test if there is a relevant difference in the average increase or decrease in activity for the daily steps taken in the month after a recommendation compared with the control group that does not receive this recommendation.

Additionally, measuring phone usage allows us to identify active users of health apps, allowing for comparison with a group that does not use health apps.

#### SO2: Correlation of Short- and Long-Term Measures

By selecting a 4-month study uptime at minimum, we can research if short-term measures (ie, activity or health app usage 1 week after the intervention) correlate with long-term measures (ie, activity a month after the intervention). To examine long-term measures further, we will attempt to monitor participants after the initial 4 months.

#### SO3: Personalization of App Allocation

To test the effect of the personalized allocation as described above, we will create, based on the collected data, a model that aims to predict which eHealth app is the most successful for which participant. The results of this model will be used to allocate apps (to intervene), and this will allow us to compare the effectiveness of randomly selected apps with those selected based on the prediction model.

### Allocation of Interventions

**Figure 2:**
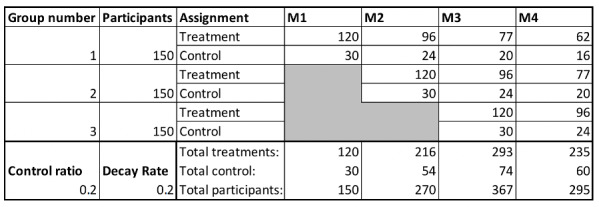
Panel size over time, including recruitment of participants, as well as the number of assigned participants in the treatment and control groups. We use a control ratio of 0.2 and a monthly decay rate of 0.2.

During the study, participants will receive recommendations to download and use certain eHealth apps. Figure 2 shows the recruitment and dropout of participants over time. We aim to recruit 150 participants each month and expect a dropout of 20% of participants each month. We explain how these groups are used for the allocation of interventions below.

#### One-Month Blocks

The study is divided into blocks of 1 month. Upon entering the panel, every participant has their activity recorded and is asked about their mood for a month, without receiving an intervention. The data serve as baseline data for participant activity. Note that the recruitment is done over the course of 3 months. From the second block onwards, participants will receive interventions. Participants will receive one intervention per block. Participants who get recommended the same app for multiple months in a row, will receive a message explaining that they should keep using the app they were assigned last month.

#### Rollout

The recruitment for Health Telescope is planned to take 3 months, enrolling 150 participants per month. Participants recruited in later months will receive interventions based on the data already generated by the existing participants.

#### Treatment and Control Group Allocation

In this section, we describe which participant at what point in time will receive which treatment (ie, which eHealth app will be recommended according to which logic).

First, in each month (Figure 2), a number of participants will be allocated to the control group; for these participants, no app will be recommended that month.

Allocation to the control group is random. For each participant, there is a 20% chance to be assigned to the control group in a specific month. Control groups are individually created for every round of intervention. Note that we choose to create control groups individually per month as opposed to a fixed control group that never receives any app recommendations throughout the study, since we believe never assigning any app may negatively impact participant engagement with the panel and may lead to a higher dropout rate.

Second, in the different months of the duration of the study, different allocation schemes will be used to recommend one of the four eHealth apps included in the study. If one of the four apps is recommended, users end up in the treatment group that specific month. We will use the following logic to allocate apps to participants: (1) In months 1 and 2, simple random allocation will be used. Thus, each app has a respective 20% chance of being selected for a participant; (2) In month 3, we will use the data collected in months 1 and 2 to create a model that aims to predict the effect of each app, for a given user, on the number of steps taken that month. We will use Bayesian additive regression tree (BART) to generate these predictions. BART is essentially a sum-of-trees model that can be used to effectively model nonlinear main and multiple-way interaction effects. Thus, it is well suited to model the interactions between the participant characteristics and the effects of the different apps. In month 3, for each new participant that is in the treatment group, the app that has the highest predicted effect will be selected. Thus, in this group of 120 participants, we will test the effects of personalizing the selected app based on the data collected previously.

### Data Collection

Data collection consists of several components including data collection during the setup phase (Figure 1) and data collection during the duration of the panel (Table 1). In this section, we describe each step and its components in turn.

**Table 1:** Summary of data collected in Health Telescope.

Data type	Frequency	Example
Step count	Every hour, the number of steps taken during that hour is measured.	2 PM-3 PM: 739 steps
Heart rate	Heart rate is measured at 1-hour intervals.	2 PM: 73 bpm
Sleep data	The start and end times of sleep are measured daily, yielding the total sleep time.	February 23: 11:16 PM-7:12 AM
Experience sampling	Up to once per day, we ask participants brief questions using push messages.	February 23, 2 PM: I generally feel energetic: Yes I currently feel happy: Completely agree Smiley: Happy
GPS location	Every 4 hours, the GPS location is saved	2 PM: 38.8977 N, 77.0365 W
Phone usage	We measure screen time and usage of apps on participants’ mobile phones. *Note: We only measure the duration of use and do not in any way measure what happens within an app.*	Chrome: 2:03 PM-2:04 PM Facebook: 2:04 PM-2:17 PM Messages: 2:23 PM-2:25 PM Mail: 2:25 PM-2:37 PM

#### Introduction Survey

The intake survey (Multimedia Appendix 1 [22]) consists of the following three parts: (1) a section providing demographic information on participants; (2) a section giving details on how active participants perceive to be; and (3) a section measuring the following constructs:

- Big Five Personality traits: A 50-item five-point Likert Big Five Personality traits questionnaire [23] measures conscientiousness, openness to experience, extraversion, agreeableness, and neuroticism. These traits have been found to correlate with physical activity and obesity [24].
- Need for Cognition scale: An 18-item seven-point Likert Need for Cognition questionnaire [25] measures the extent to which an individual is inclined towards effortful cognitive activities [26]. Need for Cognition has been found to moderate the responses individuals have toward different ways of being messaged [27].
- Susceptibility to Persuasion scale: A 26-item seven-point Likert Susceptibility to Persuasion questionnaire [12] separates an individual’s persuadability in five constructs (reciprocity, scarcity, authority, commitment, and liking). This gives direct information on how to apply the interventions in the study.

#### Wearable

Wearables have increasingly been used in research to detect daily steps taken, heart rate, sleep behavior, and even skin conductance, but show large inaccuracy in most data types [28,29]. The exception to this has been steps taken [28], which shows accuracy similar to that for devices designed solely for counting steps. Among the currently available wearables, Xiaomi MiBand 2 provides accurate measurements [28] at a relatively low price point. Furthermore, the MiBand is IP-67 waterproof and has a charge time of around 3 hours for 1-month charge. Participants are not asked to wear the MiBand for any specific number of hours or specific activity, instead participants are asked to wear the band similarly to how they would if they were not in the panel. This is done to (1) minimize participant burden and (2) maintain participant autonomy. Additionally, the MiBand allows for the transmission of unprocessed data to our servers, without sending the data to the developers first. This is a feature not present on every wearable on the market but is necessary for smooth data collection. The MiBand measures the following:

- Steps: The steps taken are measured every minute by the gyroscope in the MiBand.
- Heart rate: By default, the MiBand only measures and records heart rate when users manually do so. We changed that to also sample heart rate every hour.
- Sleep data: The gyroscope in the MiBand combined with an information layer (using rules such as “first daily unlock”) allows us to assess sleep.

#### Health Telescope App

**Figure 3:**
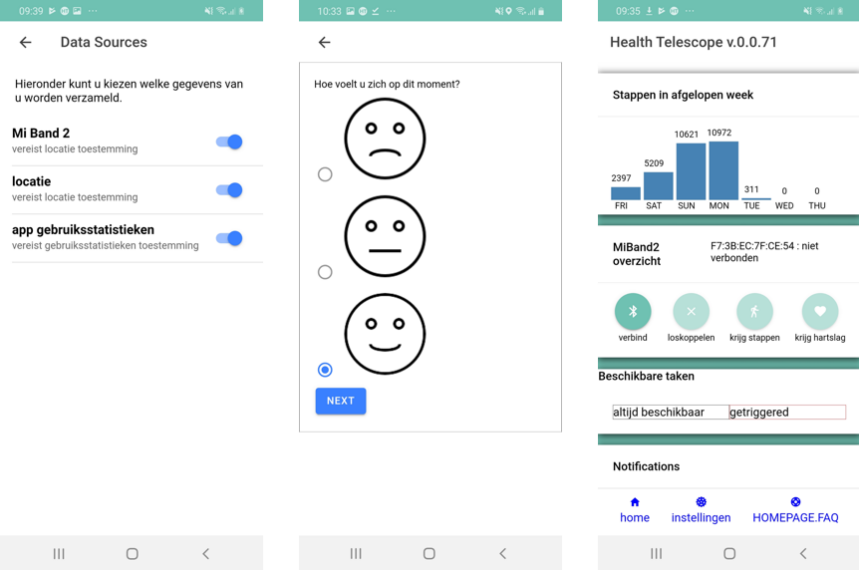
Screenshots of the Health Telescope app.

The Health Telescope app is a mobile app (screenshots of which can be seen in Figure 3) developed for the project that encompasses several goals. The app was designed to allow us to do the following:

- Communicate with participants: The app allows us to send users push messages with updates about the panel, is used to deliver interventions, and functions as a primary method of communication.
- Collect phone data: The app allows us to send the data as described in the section “Data Collection” from the users’ phones to our database.
- Record surveys: Surveys will be sent out and answered through the app.
- Manage user consent: Users can manage their accounts, including changing which data can be collected, looking into the data, and granting or revoking consent to researchers to process the data.

The app pairs with the wearable, recording steps, heart rate, and sleep data and displaying the first two to the user. The home screen further contains a list of the communications sent to the user. This consists of questionnaires and intervention messages. Every data type seen in Table 1 is collected and sent to the Health Telescope database every hour. The following data are collected by the app:

- GPS: Every hour, the GPS location of the participant’s phone is determined in the Health Telescope app. This location is first encrypted and then transmitted from the phone to the database. GPS tracking provides information regarding user behavior that might not be obtainable from activity data. Examples include commuting distance and capturing the difference between activity on a treadmill from that out in the world.
- Phone usage statistics: Every hour, a list of services that have been active on the participant’s phone will be sent to the database. This list contains the total time, total screen time, and total background time of app use. The data are used both for understanding the user’s behavior through the types of apps used and for measuring the use of a recommended eHealth app.
- Experience sampling: Participants will be given questionnaires daily, through the Health Telescope app. We use the ESM to periodically ask participants about their mood and happiness. The ESM has been shown to be a cost-effective way of measuring mental health [30]. The questions are designed as short and simple, as this can reduce participant burden [31]. By regularly and briefly checking up on how participants feel, we can obtain information on how activity relates to happiness, as well as investigate the effects of interventions on participants’ short- and long-term happiness. Two questionnaires are administered. The first is the two-item mood questionnaire. Mood will be assessed using a two-item mood questionnaire that splits mood into valence and activity. Each item is rated on a five-point Likert scale. Mood is deliberately chosen as a measure over well-being or long-term happiness, as researchers from the Tilburg Experience Sampling Center recommended it, with the main merit being its quick fluctuation; in contrast, well-being and happiness often tend to mostly change over longer periods [32], providing less information on day-to-day changes in mood. The second is the three-point smiley face questionnaire. Using smiley faces as response options is a simple technique that is argued to be an elegant way of measuring hedonic levels of happiness [33]. This coincides with the goal of measuring “in-the-moment” emotions. The response options for this questionnaire are a happy, neutral, and sad smiley face.

**Figure 4:**
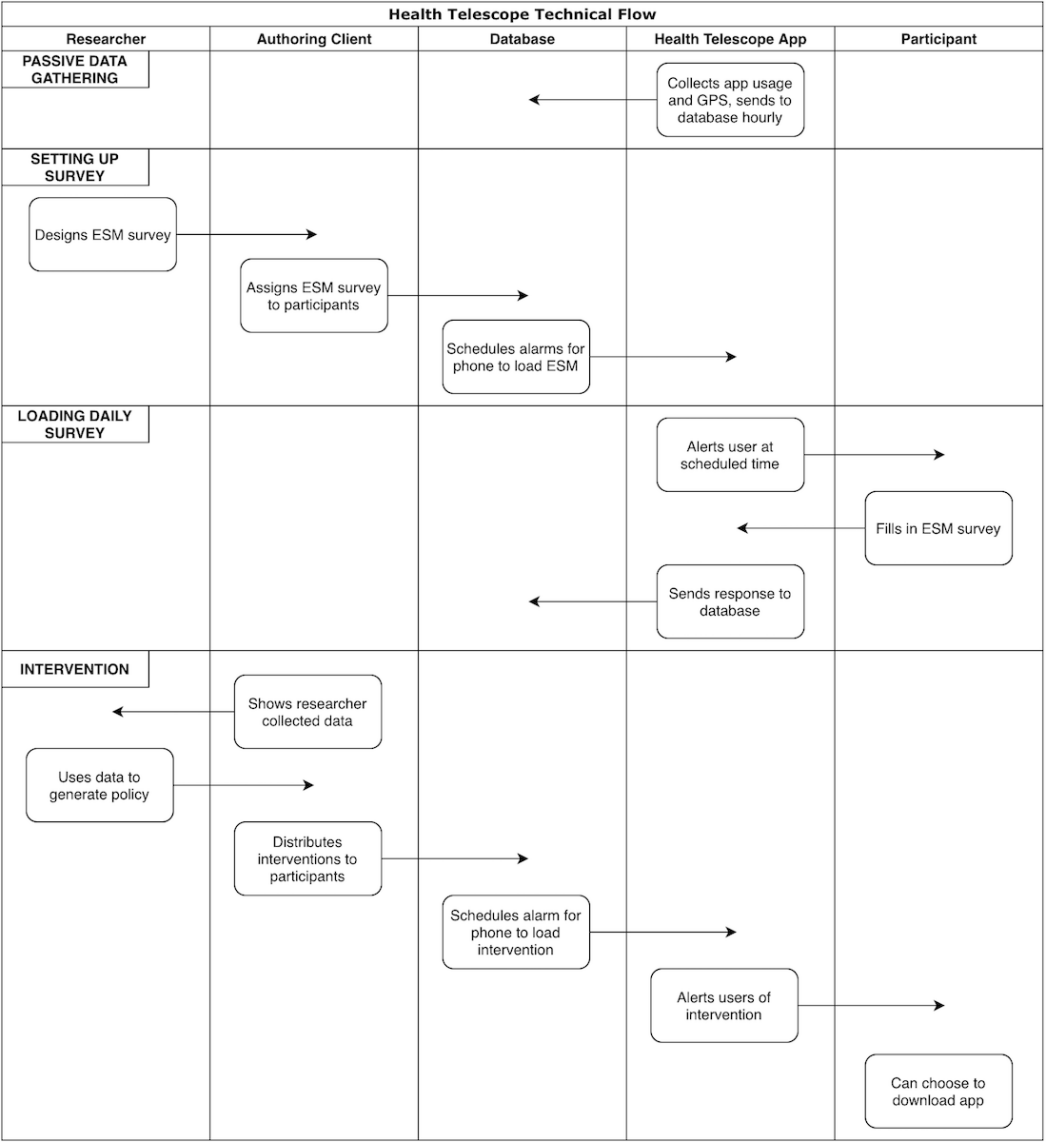
Visualization of the interactions in the panel infrastructure for passive data gathering, setting up surveys, loading of daily surveys, and interventions. ESM: experience sampling method.

Figure 4 shows the activities that take place during data collection. The data types and system in this figure are described in the sections “Data Collection” and “Data Management,” respectively. Passive data gathering happens hourly, surveys are set up by researchers to load onto participants’ phones periodically, and interventions take place monthly.

### Interventional Apps

The apps that we use for interventions are shown in Table 2. These apps were selected from a Dutch app store, the GGD App Store [34]. The *Gemeenschappelijke Gezondheidsdienst (GGD)*, the Dutch community health service, created this app store, where they test mobile apps for user-friendliness, reliability, substantiation, and privacy before being allowed on the app store. A global score, as well as a detailed verdict per category, can be seen on the website. Hence, all the apps we selected have been extensively checked. The GGD furthermore details whether the apps make use of distinct behavior change theories [35].

It is important to note that nonusage of a recommended app is important information as well. As we aim to evaluate existing eHealth apps, seeing whether they are even downloaded and used is important in its own right. As such, participants are fully allowed to not follow a recommendation if they do not believe the recommendation suits them, and participants are allowed to uninstall or stop using a recommended app at any time.

In selecting suitable apps to include in the study, we aimed to select a set of apps that are individually diverse enough to allow different user groups to find an app that might be suitable for them. This means apps should be accessible for new users who may be inexperienced with exercise and should offer content suitable for experienced users as well. Furthermore, we aimed to select apps that implement different behavior change approaches, such that we can use these approaches as one of the building blocks of our personalization approach. Ensuring that the apps used in the study are accessible and use different behavior change methods is necessary to answer the project’s objectives (PO1-3).

**Table 2:** Details of the apps used.

App name	Ways of encouragement	Behavioral change techniques
Runtastic	This app allows users to choose a level of encouragement during workouts. Results can be shared with friends.	Feedback on behavior Social comparison
Runkeeper	This app allows for goal setting. The app sends reminders to work out. Users are rewarded with badges.	Goal setting Social incentive
Human	This app incentivizes users to move for half an hour per day. Results can be shared on Facebook.	Goal setting Social comparison
Seven	The app focuses on having daily workouts and allows users to compare their performance to peers.	Commitment Social comparison

### Statistical Analysis

To be able to meet the study objectives, it is important to include sufficient participants in the study. Here, we detail in which ways the study objectives can be tested and we calculate the number of participants needed for each of these tests. Although we will conduct analyses additional to those described here to fully understand the collected data, this section aims to (1) motivate the primary analysis carried out for each study objective and (2) motivate that, given reasonable assumptions regarding the effect sizes of the selected eHealth apps, our sample size is sufficient to attain our objectives.

#### SO1: Effect of eHealth Usage

For each recommended app, a between samples *t* test comparing the average difference in activity between the group of participants who downloaded the recommended app (denoted as T) and the control group (denoted as C) will be carried out (Figure 2) in a given month. Using an estimated standard deviation of 2295 steps (as found in earlier work [36]) for activity, we will test for a difference in steps by about 1500 steps per day, which we deem a relevant and meaningful effect size. Using a power of 0.8 and a significance level of .05, we found that we need approximately 41 participants per app to conduct each of the different independent tests. Hence, our recruitment plan in which over 250 people are recruited should suffice to obtain a control group and four treatment groups (one for each app) that are large enough to meaningfully test the null hypothesis that, on average, eHealth usage increases monthly activity levels.

#### SO2: Long- and Short-Term Effects of eHealth Usage

To test whether short-term measures of physical activity meaningfully relate to long-term measures of users’ mood, we will examine the correlation between these different measures at different points in time. Our setup (Figure 2) allows us to correlate activity in the first week of usage of a recommended app to mood in week 4. We test for a correlation of 0.4 or higher, which we deem a relevant and meaningful positive correlation. Using a power of 0.8 and a significance level of .05, we found that we need approximately 235 participants to reject the null hypothesis. Our recruitment plan provides within-subject data for more than 300 subjects, and hence, we collect a sufficiently large sample to reach our objective.

#### SO3: Personalizing the Selection of eHealth Apps

The effect of using participant data to allocate interventions will be tested by analyzing the difference in activity in different intervention groups. In Figure 2, we denote different groups of participants who are recruited in different “waves.” The first two waves will allow us to collect data that we can use to create a model to predict the effectiveness of different interventions for different participants (see the section “Treatment and Control Group Allocation”). We will create a model using the data collected from 300 participants included in the first two waves of the study. Figure 2 shows the 120 interventions given in month 1 and 216 interventions in month 2, which combine to a projected data set of 336 rows to train the model. This model will be used to recommend eHealth apps in the third month, based on the best predicted change in activity for a participant; thus, effectively, our recommendations will be personalized. To test if personalization is effective, we will again conduct a between samples *t* test, and this time, we will compare the average activity of the “personalized intervention” group (group 3, treatment in Figure 2) to the control group. Similar to the analysis provided above for SO1, we found that our 120 participants in the treatment group in month 3 will be sufficient to conduct this test.

### Ethical Approval

This project has been approved by the Ethical Review Board of Tilburg University, the Netherlands. METCBrabant carried out a medical-ethical review and deemed the study to not need additional medical-ethical regulations. In this section, we will (1) detail our recruitment and information materials; (2) outline the process of ethical approval; and (3) discuss our considerations to submit for ethical approval as opposed to medical-ethical approval. We expand on the issues relating to the ethical and legal approval process, as several researchers we spoke to in this process emphasized how unique and interesting the Health Telescope is owing to the scope of the study as well as the design considerations regarding the recently changed European privacy laws. We hope that by sharing our experiences, others can more easily proceed through the required checks for similar projects.

#### Minimizing Participant Burden

To ensure ethical integrity, we have communicated our goals with the ethics review board of Tilburg University from the start of the project. We were guided in minimizing participant burden by making as many aspects of the study as possible voluntary in nature. To participate, it is not mandatory to wear the wearable, participate in surveys, download the interventional apps, or engage with these apps. This approach upholds academic ethical standards, and the optional engagement design also closely mimics real-world scenarios where users are not bound by rules or rewards to keep engaging with interventional apps and instead rely on their willpower and motivation. This allows close inspection of the effect that personalization has on engagement, activity, and well-being.

#### Ethical Versus Medical Approval

Academic research in the Netherlands needs to be reviewed and approved by an ethical review board. Additionally, the country maintains a stricter approval process when research is considered medical [37]. To belong in this category, the study needs to (1) involve *medical research* and (2) subject participants to specific actions and behavioral rules, where *medical research* is defined as “research with the goal of answering a question in the area of disease and health (etiology, pathogenesis, phenomena/symptoms, diagnosis, prevention, and result of treatment of a disease) by systematically gathering and studying data. The research aims to contribute to medical knowledge that also holds for populations outside of the direct research population.”

Health Telescope falls within a grey area of these criteria. It concerns health behaviors but does not force any rules on the participants. The Health Telescope study design has been assessed by METCBrabant [38] and approved as nonmedical-ethical research. This can be attributed to the voluntary nature of every aspect of the panel. Panel members have the choice to act on any recommendation, and data collection (as detailed in the section “Data Collection”) is optional for every data type.

### GDPR Compliance

On April 14, 2018, the GDPR [39] was adopted. Under this legislation, every entity dealing with data within the European Union has to comply with a set of data rights aimed to give the group of users, formalized as *data subjects*, more rights over the data they produce. The Health Telescope project was designed to comply with this new regulation. Under the GDPR, data collection and storage need to be done in a way that puts data subjects in control of their data, and privacy is ensured by design. The GDPR officer of Tilburg University checked the study’s alignment with the GDPR principles and approved the study. Since this law is novel, we devote special attention to the actions taken when setting up Health Telescope to comply with GDPR regulations. We discuss each identified right and subsequently detail how our study setup complies. The eight rights that data subjects have, as well as the way we implement these rights, are as follows:

1. Right to be informed: Data subjects have the right to be informed about the processing of their data, as well as any changes to the data made due to any of the other rights. We comply with this right by design, by informing participants of what data are collected and why this is useful, sharing the results from the study, and keeping an open line of communication in case any information is not clear using the Health Telescope app.
2. Right to access: Data subjects have the right to, at any time, ask whether their data are being processed for any reason and, if so, request access to their data. This right is included in Health Telescope. The way data are processed is communicated from the start of the study through the information received by participants. Additionally, participants can request access to their data at any time by sending an email to the Health Telescope team. These requests are processed within 4 weeks.
3. Right to data portability: Data subjects have the right to, at any time, transfer their data from one digital processing system to another. We incorporate this right similarly to the right to access. Participants can get a full copy of their data and can receive extracts from our server in CSV files by sending an email request.
4. Right to restrict processing: Data subjects have the right to restrict the processing of their data to the procedures they consent to. This right is included in our design in two ways. First, participants can choose whether they want data to be recorded for any data type separately, and they can change their consent on this at any time. Second, if additional research is done through Health Telescope, participants can choose to opt-in or out of this research.
5. Right to object: Data subjects have the right to object to any processing of their personal data. The combination of implementing right 2 and right 4 leads to compliance with this right. The use of communication channels combined with participants’ ability to have their data erased ensures compliance.
6. Right to rectification: Data subjects have the right to have any incorrect data of theirs that is stored be rectified by the data controller. Through the implementation of the right to access and clear communication, we aim to give participants the tools that also ensure this right is respected. If participants want any incorrect data to be rectified, it will be rectified.
7. Right to erasure: Data subjects have the right to, when data is processed with their consent as a legal basis, withdraw this consent and have all personal data deleted entirely. We include this right in our design. Participants have the ability to delete their data irreversibly.
8. Right to not be subject to automated processing: Data subjects have the right to not be subject to decisions made purely from automated processing. This includes profiling, which produces legal effects greatly affecting them. This specific right has three exceptions to it [40]. For the interventions in Health Telescope, we need to automatically process data into a set of rules. One of the exceptions to this right is informed consent. If participants are aware that their data can be used for the interventions and consent to it, we can use automated processing.

Transmission of data between devices uses HTTPS encryption, and all data are kept in encrypted storage. Servers can only be accessed after user authorization, and all server access is logged. In case of a data leak, we will follow the protocol for data leaks as defined by Tilburg University [6,39,41].

### Dissemination

#### Publishing

The research findings following the Health Telescope study will be presented at international conferences, as well as reported in international peer-reviewed academic journals. Other longitudinal panel studies have increased the scientific output from their research by allowing additional researchers to analyze the panel data. We aim to follow this procedure. The Health Telescope team is actively open to cooperation with organizations interested in the data generated in the study. Applying for data access or cooperation will be possible through the Health Telescope website.

#### Data Management

The Health Telescope app and database were created by RoboticBit. The database can be accessed by researchers through an authoring client, a web interface with tools for researchers to set up surveys, distribute interventions, and manage participants. The database will be realized and managed on the network of Technical University Eindhoven. Participants can request access to their data by sending an email to the Health Telescope team. The primary researchers of the Health Telescope team will have full access to the database. Any additional researchers who would like to access the data can request extracts of the part of the data that they need.

#### Data Sharing

During the panel study and for a maximum of 10 years after the data collection has concluded, we aim to give researchers access to the collected data. There is a procedure to access the data based on explicit consent from participants, as the data are very sensitive.

## Results

Funding for the project began in November 2017. Ethical approval was obtained in February 2019. Privacy and data handling were investigated and approved in a data protection impact assessment performed by Technical University Eindhoven in April 2019. The data collection software has been developed, and an internal pilot was held in June 2019. Another pilot was planned for Q2 of 2020 but has been postponed owing to COVID-19 quarantine measures. We currently expect to start recruitment in Q4 of 2020. Initial results will be published in 2021, and the full study will take 4 months.

## Discussion

### Revisiting the Objectives

The Health Telescope study aims to investigate how different individuals respond to different ways of being encouraged to increase their physical activity using eHealth apps. The study was specifically designed to allow for long-term data collection as the long-term effects of the use of eHealth apps are largely unknown. This long-term data collection allows us to satisfy our first goal (SO1) of estimating the long-term effects of eHealth interventions and allows us to passively collect enough data to accomplish the second goal (SO2) of comparing short- and long-term outcomes. Finally, our interventions allow us to test whether a personalized selection of eHealth apps improves their effectiveness (SO3). Note that we have specifically selected apps using different behavioral change techniques. This allows us to not only simply test the effect of the app but also compare how different techniques influence a person.

We hope that our thorough description of the process of setting up this panel study is able to accomplish the objectives of this paper. To accomplish the first objective (PO1), we detailed the panel setup, explaining both the process and reasoning for our decisions. We described the process of being in the panel, showed the collected data, and included the steps for data access to accomplish the second objective (PO2) and help future researchers who want to use Health Telescope data. Finally, we detailed the design, approval procedures, and plans for analysis to achieve the third objective (PO3) and guide researchers interested in setting up similar research.

### Limitations

One aspect of the study that carries risk is the potential loss of data that may come with the power that Health Telescope participants have over their data. While the information supplied to participants can help them understand why the collection of data is important, their preferences toward privacy might hinder data collection. To limit this risk, we have performed a pilot assessment focused on user experience and have made some alterations to the app based on the results of this pilot. Additionally, as recruitment is gradual, we can observe participant behavior and investigate any risks during recruitment. Another factor that is difficult to estimate is the dropout rate in the panel. While measures to minimize the consequences of dropout have been put in place, such as a plan to keep participants engaged, periodic recruitment to keep an active panel, a panel size allowing for some dropout, and an iterative study design, it is important to closely monitor dropout.

A potential confounding factor in our setup is the fact that the Health Telescope app itself provides minimal feedback regarding the activity of the participant. While this might influence the behavior of participants, not showing such feedback was deemed not feasible in pretests as participants wondered whether their MiBand was properly paired. However, given that our final comparisons are experimental and the feedback the Health Telescope app provides is the same in the treatment and control arms, we believe that this minimal feedback does not interfere with our objectives.

Panel studies are known to have several types of biases, including attrition bias of dropout, nonresponse bias, selection bias, and participation bias. These biases could distort the results if they lead to a homogeneous participant group. To limit this, recruitment is performed through multiple channels, and gradual recruitment. Close monitoring for participant group consistency can help shape the desired diverse group of participants.

### Conclusions

This protocol described the setup of the Health Telescope study. Health Telescope will enable us to answer several pressing questions regarding the long-term effectiveness of eHealth apps designed to motivate users to lead an active lifestyle. Furthermore, the longitudinal nature of the study, combined with the unique ability to provide interventions over time, will allow us to study the effects of personalizing eHealth recommendations. We hope that by sharing this protocol, we can make it easier for others to (1) analyze the data resulting from the Health Telescope study, which we aim to disclose, and (2) set up their own longitudinal evaluations of the effectiveness of eHealth apps.

## Appendix

Multimedia Appendix 1 Introduction survey.

## Abbreviations

BART: Bayesian additive regression tree
ESM: experience sampling method
GGD: Gemeenschappelijke Gezondheidsdienst
GDPR: General Data Protection Regulation
PO: protocol objective
SO: study objective
