# Supplementary material for: Developing Effective Methods for Electronic Health Personalization: Protocol for Health Telescope, a Prospective Interventional Study
Source: JMIR Res Protoc. 2020 Jul 31;9(7):e16471. doi: 10.2196/16471 (PMC7428913; doi:10.2196/16471)
Supplement: Multimedia Appendix 1 [file resprot_v9i7e16471_app1.docx]

# Appendix A – Introduction Survey

In this appendix, we detail the questions that are asked in the introduction survey that participants fill in during recruitment. Aside from question 1, no question is mandatory to fill in. During recruitment, users are instructed to fill in the questionnaire, but explicitly to not any question they do not wish to answer. The questionnaire consists of four parts: background (10 questions); need for cognition (18 questions); big five inventory (44 questions); need for cognition (12 questions).

### Part I – Background questions

The first part of the introduction survey contains questions regarding the participants’ background, such as education, income, and food/smoking behaviors. For all questions except for e-mail address, participants are able to not answer the question. The table below shows the questions as they appear in the survey, in Dutch; an English translation of the question, added to the protocol for clarification, and a list of response categories, where this applies.

| **Question** | **Description in survey (Dutch)** | **Question in English** | **Categories response** |
| --- | --- | --- | --- |
| 1 | Wat is uw e-mailadres? | What is your e-mail address? | N/A |
| 2 | Wat is uw leeftijd in jaren? | What is your age in years? | N/A |
| 3 | Wat is uw hoogste voltooide opleidingsniveau? | What is your highest completed level of education? | [Prefer not to answer;  Primary school;  High school;  MBO degree;  Applied university degree;  Bachelor’s degree;  Master’s degree;  Ph.D.] |
| 4 | Wat is uw bruto jaarlijks inkomen in Euro? | What is your gross yearly income in Euro? | [Prefer not to answer;  1-9,999;  10,000-19,999;  20,000-29,999;  30,000-39,999;  40,000-49,999;  50,000-59,999;  60,000-69,999;  70,000-79,999;  80,000-89,999;  90,000-99,999;  100,000 or more] |
| 5 | Wat is uw geslacht? | What is your gender? | [Prefer not to answer;  Male;  Female;  Other] |
| 6 | Wat is uw burgerlijke staat? | What is your marital status? | [Prefer not to answer;  Married;  Divorced;  Widowed;  Never married] |
| 7 | Welk van de volgende beschrijft uw werksituatie het best? | Which of the following describes your employment most accurately? | [Prefer not to answer;  Employed, working 1-39 hours per week;  Employed, working 40 or more hours per week;  Not employed, looking for work;  Not employed, NOT looking for work;  Retired;  Disabled, not able to work] |
| 8 | Rookt u? | Do you currently smoke? | [Prefer not to answer;  Yes, daily;  Yes, once per week;  No, I quit;  No, I have never used tobacco] |
| 9 | Hoe vaak doet u aan sport? | How often do you engage in sports? | [Prefer not to answer;  Daily;  3-5 times per week;  1-2 times per week;  Rarely] |
| 10 | Welke van de onderstaande opties beschrijft uw diëetgewoonten het best? | Which of the following best describes your dietary habits? | [Prefer not to answer;  Vegetarian;  Vegan;  Glutenfree;  No restrictions] |

### Part II – Need for Cognition

The second part of the survey contains an 18-item Need for Cognition test. Every response is a 7-point Likert scale, ranging from ‘Fully disagree’ (1) to ‘Fully agree’ (7). The table below shows the questions asked in Dutch, and the original questionnaire in English, added to this protocol for clarification.

| **Question** | **Description in survey (Dutch)** | **Question in English** |
| --- | --- | --- |
| 1 | Ik heb liever te maken met complexe dan eenvoudige problemen | I would prefer complex to simple problems. |
| 2 | Ik vind het fijn om een situatie aan te pakken die veel nadenken vereist | I like to have the responsibility of handling a situation that requires a lot of thinking. |
| 3 | Nadenken is niet mijn idee van plezier hebben | Thinking is not my idea of fun.* |
| 4 | Ik doe liever iets dat weinig denkwerk vereist dan iets waarbij ik veel na moet denken | I would rather do something that requires little thought than something that is sure to challenge my thinking abilities.* |
| 5 | Ik probeer te anticiperen en situaties te vermijden waarin de kans groot is dat ik ergens diep over moet  nadenken | I try to anticipate and avoid situations where there is likely a chance I will have to think in depth about something.* |
| 6 | Ik vind het leuk om veel en lang te overleggen | I find satisfaction in deliberating hard and for long hours. |
| 7 | Ik denk maar zo hard na als nodig is | I only think as hard as I have to.* |
| 8 | Ik denk liever aan kleine, dagelijkse projecten dan aan langetermijnprojecten | I prefer to think about small, daily projects to long-term ones.* |
| 9 | Ik hou van taken waar ik weinig bij na hoef te denken als ik ze eenmaal heb geleerd | I like tasks that require little thought once I’ve learned them.* |
| 10 | Het idee de top te bereiken door op gedachten te vertrouwen, spreekt me aan | The idea of relying on thought to make my way to the top appeals to me. |
| 11 | Ik geniet echt van een taak waarbij ik nieuwe oplossingen voor problemen bedenk | I really enjoy a task that involves coming up with new solutions to problems. |
| 12 | Het leren van nieuwe manieren van denken, maakt me niet erg enthusiast | Learning new ways to think doesn’t excite me very much.* |
| 13 | Ik heb graag dat mijn leven gevuld is met puzzels die ik moet oplossen | I prefer my life to be filled with puzzles that I must solve. |
| 14 | Abstract denken spreekt me erg aan | The notion of thinking abstractly is appealing to me. |
| 15 | Ik heb liever een taak die intellectueel, moeilijk en belangrijk is dan een taak die enigszins belangrijk is maar niet veel aandacht vereist | I would prefer a task that is intellectual, difficult, and important to one that is somewhat important but does not require much thought. |
| 16 | Ik voel opluchting in plaats van tevredenheid na het voltooien van een taak die veel mentale inspanning nodig had | I feel relief rather than satisfaction after completing a task that required a lot of mental effort.* |
| 17 | Het is genoeg voor mij dat de klus geklaard is; het maakt me niet uit hoe of waarom het werkt | It’s enough for me that something gets the job done; I don’t care how or why it works.* |
| 18 | Ik denk meestal veel na over problemen, zelfs als ze mij niet persoonlijk raken | I usually end up deliberating about issues even when they do not affect me personally. |

### Part III – Big Five Inventory

The third part of the survey contains a 44-item Big Five Inventory questionnaire. Every response is a 5-point Likert scale, ranging from ‘Fully disagree’ (1) to ‘Fully agree’ (7). The table below shows the questions asked in Dutch, and the original questionnaire in English, added to this protocol for clarification.

| **Question** | **Description in survey (Dutch)** | **Question in English** |
| --- | --- | --- |
|  | Ik zie mezelf als iemand die… | I see myself as someone who… |
| 1 | ...Spraakzaam is | …Is talkative |
| 2 | ...Geneigd is kritiek te hebben op anderen | …Tends to find fault with others |
| 3 | ...Grondig te werk gaat | …Does a thorough job |
| 4 | ...Somber is | …Is depressed, blue |
| 5 | ...Origineel is, met nieuwe ideeën komt | …Is original, comes up with new ideas |
| 6 | ...Terughoudend is | …Is reserved |
| 7 | ...Behulpzaam en onzelfzuchtig ten opzichte van anderen is | …Is helpful and unselfish with others |
| 8 | ...Een beetje nonchalant kan zijn | …Can be somewhat careless |
| 9 | ...Vol energie is | …Is full of energy |
| 10 | ...Snel ruzie maakt | …Starts quarrels with others |
| 11 | ...Een werker is waar men van op aan kan | …Is a reliable worker |
| 12 | ...Gespannen kan zijn | …Can be tense |
| 13 | ...Scherpzinnig, een denker is | …Is ingenious, a deep thinker |
| 14 | ...Veel enthousiasme opwekt | …Generates a lot of enthusiasm |
| 15 | ...Vergevingsgezind is | …Has a forgiving nature |
| 16 | ...Doorgaans geneigd is tot slordigheid | …Tends to be disorganized |
| 17 | ...Een levendige fantasie heeft | …Has an active imagination |
| 18 | ...Doorgaans stil is | …Tends to be quiet |
| 19 | ...Mensen over het algemeen vertrouwt | …Is generally trusting |
| 20 | ...Geneigd is lui te zijn | …Tends to be lazy |
| 21 | ...Emotioneel stabiel is, niet gemakkelijk overstuur raakt | …Is emotionally stable, not easily upset |
| 22 | ...Vindingrijk is | …Is inventive |
| 23 | ...Voor zichzelf opkomt | …Has an assertive personality |
| 24 | ...Koud en afstandelijk kan zijn | …Can be cold and aloof |
| 25 | ...Humeurig kan zijn | …Has an active imagination |
| 26 | ...Waarde hecht aan kunstzinnige ervaringen | …Values artistic, aesthetic experiences |
| 27 | ...Soms verlegen, geremd is | …Is sometimes shy, inhibited |
| 28 | ...Attent en aardig is voor bijna iedereen | …Is considerate and kind to almost everyone |
| 29 | ...Dingen efficiënt doet | …Does things efficiently |
| 30 | ...Kalm blijft in gespannen situaties | …Remains calm in tense situations |
| 31 | ...Een voorkeur heeft voor werk dat routine is | …Prefers work that is routine |
| 32 | ...Hartelijk, een gezelschapsmens is | …Is outgoing, sociable |
| 33 | ...Plannen maakt en deze doorzet | …Makes plans and follows through with them |
| 34 | ...Gemakkelijk zenuwachtig wordt | …Gets nervous easily |
| 35 | ...Graag nadenkt, met ideeën speelt | …Likes to reflect, play with ideas |
| 36 | ...Weinig interesse voor kunst heeft | …Has few artistic interests |
| 37 | ...Graag samenwerkt met anderen | …Likes to cooperate with others |
| 38 | ...Gemakkelijk afgeleid is | …Is easily distracted |
| 39 | ...Het fijne weet van kunst, muziek of literatuur | …Is sophisticated in art, music, or literature |
| 40 | ...Ontspannen is, goed met stress kan omgaan | …Is relaxed, handles stress well |
| 41 | ...Benieuwd is naar veel verschillende dingen | …Is curious about many different things |
| 42 | ...Zich veel zorgen maakt | …Worries a lot |
| 43 | ...Volhoudt tot de taak af is | …Makes plans and follows through with them |
| 44 | ...Soms grof tegen anderen is | …Is sometimes rude to others |

### Part IV – Susceptibilility to Persuasion

The fourth part of the survey contains a 12-item Susceptibility to persuasion test. Every response is a 7-point Likert scale, ranging from ‘Fully disagree’ (1) to ‘Fully agree’ (7). The table below shows the questions asked in Dutch, and the original questionnaire in English, added to this protocol for clarification.

| **Question** | **Description in survey (Dutch)** | **Question in English** |
| --- | --- | --- |
| 1 | Wanneer mijn familie mij een gunst verleent, dan ben ik erg geneigd om een gunst terug te verlenen. | When a family member does me a favor, I am very inclined to return this favor |
| 2 | Ik verleen altijd een gunst terug | I always pay back a favor |
| 3 | Ik geloof dat zeldzame (schaarse) producten meer waarde hebben dan massaproducten | I believe rare products (scarce) are more valuable than mass products |
| 4 | Wanneer mijn favoriete winkel op het punt staat te sluiten, dan zou ik die bezoeken omdat het mijn laatste kans is | When my favorite shop is about to close, I would visit it since it is my last chance |
| 5 | Ik volg altijd advies op van mijn huisarts | I always follow advice from my general practitioner. |
| 6 | Wanneer een professor mij iets vertelt dan heb ik de neiging te geloven dat het waar is | When a professor tells me something I tend to believe it is true. |
| 7 | Wanneer ik een afspraak maak, dan kom ik na wat ik heb beloofd | Whenever I commit to an appointment I do as I told. |
| 8 | Ik probeer alles te doen dat ik beloofd heb | I try to do everything I have promised to do. |
| 9 | Wanneer iemand uit mijn omgeving me een goed boek aanraadt, heb ik de neiging dit boek te lezen | If someone from my social network notifies me about a good book, I tend to read it. |
| 10 | Wanneer ik me in een nieuwe situatie bevind, kijk ik naar anderen om te zien wat ik moet doen | When I am in a new situation I look at others to see what I should do. |
| 11 | Ik accepteer advies van mijn omgeving | I accept advice from my social network. |
| 12 | Wanneer ik iemand aardig vind, ben ik meer geneigd hem/haar te geloven | When I like someone, I am more inclined to believe him or her. |
